# Tobacco Use and Tobacco-Related Behaviors — 11 Countries, 2008–2017

**DOI:** 10.15585/mmwr.mm6841a1

**Published:** 2019-10-18

**Authors:** Indu B. Ahluwalia, René A. Arrazola, Luhua Zhao, Jing Shi, Anna Dean, Edward Rainey, Krishna Palipudi, Evelyn Twentyman, Brian S. Armour

**Affiliations:** ^1^Office on Smoking and Health, National Center for Chronic Disease Prevention and Health Promotion, CDC; ^2^CDC Foundation, Atlanta, Georgia.

Each year, tobacco use is responsible for approximately 8 million deaths worldwide, including 7 million deaths among persons who use tobacco and 1.2 million deaths among nonsmokers exposed to secondhand smoke (SHS) (*1*). Approximately 80% of the 1.1 billion persons who smoke tobacco worldwide reside in low- and middle-income countries (*2*,*3*). The World Health Organization’s (WHO’s) Framework Convention on Tobacco Control (FCTC) provides the foundation for countries to implement and manage tobacco control through the MPOWER policy package,* which includes monitoring tobacco use, protecting persons from SHS, warning them about the danger of tobacco, and enforcing bans on tobacco advertising, promotion, or sponsorship (tobacco advertising) (*4*). CDC analyzed data from 11 countries that completed two or more rounds of the Global Adult Tobacco Survey (GATS) during 2008–2017. Tobacco use and tobacco-related behaviors that were assessed included current tobacco use, SHS exposure, thinking about quitting because of warning labels, and exposure to tobacco advertising. Across the assessed countries, the estimated percentage change in tobacco use from the first round to the most recent round ranged from −21.5% in Russia to 1.1% in Turkey. Estimated percentage change in SHS exposure ranged from −71.5% in Turkey to 72.9% in Thailand. Estimated percentage change in thinking about quitting because of warning labels ranged from 77.4% in India to −33.0% in Turkey. Estimated percentage change in exposure to tobacco advertising ranged from −66.1% in Russia to 44.2% in Thailand. Continued implementation and enforcement of proven tobacco control interventions and strategies at the country level, as outlined in MPOWER, can help reduce tobacco-related morbidity and mortality worldwide (*3*,*5*,*6*).

GATS is a nationally representative household survey of noninstitutionalized adults aged ≥15 years that uses a standard core questionnaire, sample design, and data collection methods.^†^ GATS data were analyzed from 11 countries with at least two rounds of data collection during 2008–2017. Sample sizes in the first round ranged from 5,581 (Uruguay) to 69,296 (India) and in the most recent round, from 4,966 (Uruguay) to 74,037 (India).^§^ Because Turkey is the only country to have conducted three rounds of GATS data collection (2008, 2012, and 2016), 2008 was used as the baseline and 2016 as the follow-up to allow for results to be presented similarly to other countries; however, to assess changes over time in that country, all three rounds of data from Turkey were also analyzed. Response rates in the first round of GATS ranged from 76.2% (Ukraine) to 97.7% (Russia) and in the most recent round, from 64.4% (Ukraine) to 98.2% (Russia). Data were adjusted for nonresponse through weighting to provide nationally representative estimates among persons aged ≥15 years.

The prevalence and weighted population estimates of four tobacco control indicators were calculated: 1) current tobacco use; 2) SHS exposure; 3) thinking about quitting because of warning labels; and 4) exposure to tobacco advertising. Current tobacco use^¶^ was defined as either currently smoking tobacco, currently using smokeless tobacco, or both on a daily or less than daily basis.** SHS exposure was defined as being exposed to SHS in the past 30 days in any of four public places: restaurants, government buildings, health care facilities, or public transportation.^††^ Thinking about quitting because of warning labels was defined as currently smoking tobacco and noticing health warnings on a cigarette package leading to thinking about quitting in the past 30 days.^§§^ Exposure to tobacco advertising was defined as being aware of cigarette advertising, promotions, or sponsorship in the last 30 days.^¶¶^

Country-specific prevalence and population estimates with corresponding 95% confidence intervals (CIs) were calculated for current tobacco use, SHS exposure, thinking about quitting because of warning labels, and exposure to tobacco advertising. Also, percentage point differences and percentage differences in prevalences and differences in population estimates were calculated. Z-tests were used to assess statistically significant differences (p<0.05) between surveys. All statistical analyses were conducted using SAS-callable SUDAAN (version 11.0; RTI International).

Across the 11 countries, the overall population estimate for current tobacco use decreased by approximately 20 million between GATS rounds, with estimated percentage point differences ranging from an 8.5% decline (Russia) to a 0.4% increase (Turkey) (Table). The overall population estimate for SHS exposure decreased by approximately 53.4 million, with estimated percentage point differences ranging from a 24.5% decrease (Russia) to a 13.0% increase (Thailand). The overall population estimate for thinking about quitting because of warning labels increased by approximately 12.4 million, with estimated percentage point differences ranging from a 22.1% increase (India) to an 18.2% decrease (Vietnam). The overall population estimate for exposure to tobacco advertising decreased by approximately 98.8 million, with estimated differences ranging from a 45.0% decline (Russia) to a 7.9% increase (Thailand). Analysis of the three rounds of data from Turkey showed that current tobacco use decreased during 2008–2012 and then increased during 2012–2016; thinking about quitting because of warning labels increased during 2008–2012 and then decreased during 2012–2016; SHS exposure decreased over all three rounds; and exposure to tobacco advertising did not change significantly during 2008–2012 or 2012–2016 (Figure 1). The WHO target for 2030 is a 30% reduction in current tobacco use among persons aged ≥15 years (Figure 2). From 2009 to 2016, Russia had a 21.5% reduction in the number of current tobacco users, and six countries (Bangladesh, Brazil, India, Philippines, Ukraine, and Uruguay) had reductions ranging from 13.1% to 19.9%. Two countries (Thailand and Vietnam) had reductions of <5%; Mexico and Turkey experienced slight increases. The differences in prevalence estimates and population estimates are due to changing population sizes of the countries over time. Prevalence and population estimates were included for all indicators: current tobacco use, secondhand smoke exposure, thinking about quitting because of warning labels, and exposure to tobacco advertisements in any location.

**TABLE:** Estimated prevalence and weighted population estimates* of persons aged ≥15 years of age who currently used tobacco, who were exposed to secondhand smoke, who contemplated quitting because of warning labels on cigarette packages, and who were exposed to tobacco advertisements — 11 countries, Global Adult Tobacco Survey (GATS), 2008–2017

Tobacco use category	Prevalence^†^				Population (millions)^†^		
	Baseline round	Most recent round	% Point difference	% Change^§^	Baseline round	Most recent round	Population difference
Country (yrs)	% (95% CI)				Estimate (95% CI)		
**Current tobacco use**							
Bangladesh (2009, 2017)	43.3 (41.7–45.0)	35.3 (33.9–36.7)	−8.0^¶^	−18.5^¶^	41.3 (38.9–43.6)	37.7 (36.0–39.4)	−3.5^¶^
Brazil** (2008, 2013)	18.5 (18.0–19.0)	15.0 (14.5–15.5)	−3.5^¶^	−19.2^¶^	24.6 (23.3–25.9)	21.9 (21.1–22.7)	−2.7^¶^
India (2009/10, 2016/17)	34.6 (33.6–35.5)	28.6 (27.9–29.3)	−5.9^¶^	−17.2^¶^	274.8 (260.7–289.0)	266.8 (258.1–275.5)	−8.0
Mexico (2009, 2015)	16.5 (15.3–17.8)	16.6 (15.7–17.6)	0.1	0.8	11.0 (9.3–12.7)	14.4 (13.5–15.3)	3.3^¶^
Philippines (2009, 2015)	29.7 (28.5–31.0)	23.8 (22.8–24.9)	−5.9^¶^	−19.9^¶^	18.0 (17.0–19.1)	16.5 (15.5–17.6)	−1.4^¶^
Russia^††^ (2009, 2016)	39.4 (38.0–40.8)	30.9 (29.4–32.4)	−8.5^¶^	−21.5^¶^	44.1 (41.2–47.0)	34.2 (32.5–36.0)	−9.8^¶^
Thailand (2009, 2011)	27.2 (26.2–28.3)	26.9 (25.7–28.1)	−0.4	−1.4	14.3 (13.7–14.9)	14.5 (13.8–15.3)	0.2
Turkey^§§^ (2008, 2016)	31.2 (30.0–32.6)	31.6 (30.2–33.0)	0.4	1.1	15.9 (15.2–16.7)	19.2 (18.2–20.1)	3.2^¶^
Ukraine^††^ (2010, 2016)	28.4 (27.2–29.7)	23.0 (21.8–24.3)	−5.4^¶^	−19.0^¶^	9.7 (9.2–10.2)	8.2 (7.7–8.7)	−1.4^¶^
Uruguay (2009, 2017)	25.0 (23.4–26.6)	21.7 (20.4–23.0)	−3.3^¶^	−13.1^¶^	0.6 (0.5–0.6)	0.5 (0.5–0.6)	<-0.1
Vietnam (2010, 2015)	25.2 (24.0–26.4)	24.2 (22.9–25.5)	−1.0	−4.1	16.0 (15.2–16.8)	16.3 (15.3–17.3)	0.3
**Secondhand smoke exposure**							
Bangladesh (2009, 2017)	45.0 (43.4–46.5)	34.1 (32.5–35.7)	−10.9^¶^	−24.2^¶^	42.8 (40.1–45.5)	36.2 (34.1–38.3)	−6.5^¶^
Brazil** (2008, 2013)	N/A	N/A	N/A	N/A	N/A	N/A	N/A
India (2009/10, 2016/17)	29.0 (28.1–29.9)	23.1 (22.4–23.9)	−5.9^¶^	−20.3^¶^	228.4 (216.3–240.4)	214.7 (206.5–222.9)	−13.6
Mexico (2009, 2015)	23.3 (21.5–25.1)	24.8 (23.6–26.0)	1.5	6.5	15.9 (13.5–18.3)	21.6 (20.4–22.8)	5.7^¶^
Philippines (2009, 2015)	55.0 (53.3–56.7)	37.8 (36.0–39.6)	−17.2^¶^	−31.3^¶^	33.6 (31.7–35.5)	26.3 (24.5–28.0)	−7.3^¶^
Russia^††^ (2009, 2016)	35.1 (33.0–37.3)	10.7 (9.3–12.2)	−24.5^¶^	−69.7^¶^	39.2 (35.9–42.6)	11.7 (10.1–13.4)	−27.5^¶^
Thailand (2009, 2011)	17.8 (16.7–18.9)	30.8 (29.0–32.6)	13.0^¶^	72.9^¶^	9.2 (8.5–9.8)	16.4 (15.3–17.5)	7.2^¶^
Turkey^§§^ (2008, 2016)	31.5 (29.8–33.3)	9.0 (8.0–10.1)	−22.5^¶^	−71.5^¶^	16.0 (15.0–17.0)	5.2 (4.6–5.9)	−10.7^¶^
Ukraine^††^ (2010, 2016)	29.0 (27.3–30.8)	12.5 (11.1–14.0)	−16.5^¶^	−57.0^¶^	9.9 (9.2–10.6)	4.4 (3.8–5.0)	−5.4^¶^
Uruguay (2009, 2017)	8.8 (7.8–10.0)	6.5 (5.6–7.6)	−2.3^¶^	−26.5^¶^	0.2 (0.1–0.2)	0.1 (0.1–0.2)	<-0.1
Vietnam (2010, 2015)	32.5 (31.2–33.8)	37.3 (35.9–38.8)	4.9^¶^	15.0^¶^	20.8 (19.9–21.7)	25.8 (24.6–26.9)	4.9^¶^
**Thinking about quitting because of warnings labels**							
Bangladesh (2009, 2017)	58.5 (55.1–61.7)	75.6 (71.9–78.9)	17.1^¶^	29.3^¶^	12.5 (11.5–13.5)	14.4 (13.4–15.5)	1.9^¶^
Brazil** (2008, 2013)	65.0 (63.4–66.5)	54.3 (50.3–54.2)	−10.7^¶^	−19.6^¶^	15.7 (14.7–16.6)	11.2 (10.6–11.9)	−4.4^¶^
India (2009/10, 2016/17)	28.6 (26.8–30.4)	50.7 (48.8–52.7)	22.1^¶^	77.4^¶^	31.6 (29.1–34.1)	50.4 (47.4–53.5)	18.8^¶^
Mexico (2009, 2015)	33.0 (30.1–36.0)	43.2 (39.9–46.5)	10.2^¶^	31.0^¶^	3.6 (2.9–4.2)	6.1 (5.5–6.7)	2.5^¶^
Philippines (2009, 2015)	37.4 (34.8–40.0)	44.6 (41.5–47.7)	7.2^¶^	19.4^¶^	6.4 (5.9–6.9)	7.0 (6.3–7.7)	0.5
Russia^††^ (2009, 2016)	31.7 (28.9–34.6)	36.1 (33.4–38.8)	4.4^¶^	13.8^¶^	13.8 (12.3–15.3)	12.2 (11.1–13.3)	−1.6
Thailand (2009, 2011)	67.0 (64.4–69.5)	62.6 (60.0–65.2)	−4.4^¶^	−6.5^¶^	8.3 (7.9–8.8)	8.1 (7.5–8.7)	−0.2
Turkey^§§^ (2008, 2016)	46.3 (43.6–49.1)	31.0 (28.5–33.7)	−15.3^¶^	−33.0^¶^	7.4 (6.8–7.9)	5.9 (5.3–6.4)	−1.4^¶^
Ukraine^††^ (2010, 2016)	59.7 (56.1–63.2)	54.0 (50.6–57.5)	−5.7^¶^	−9.5^¶^	5.7 (5.3–6.2)	4.4 (4.0–4.7)	−1.3^¶^
Uruguay (2009, 2017)	42.9 (39.4–46.4)	42.9 (39.4–46.6)	0.1	0.2	0.2 (0.2–0.2)	0.2 (0.2–0.2)	<0.1
Vietnam (2010, 2015)	66.7 (63.9–69.4)	48.5 (45.5–51.5)	−18.2^¶^	−27.2^¶^	10.1 (9.5–10.7)	7.5 (6.8–8.1)	−2.6^¶^
**Exposure to advertisements, promotions, or sponsorships in any location**							
Bangladesh (2009, 2017)	48.7 (46.2–51.2)	39.6 (36.7–42.5)	−9.1^¶^	−18.8^¶^	45.8 (42.5–49.1)	28.8 (26.3–31.3)	−16.9^¶^
Brazil** (2008, 2013)	N/A	N/A	N/A	N/A	N/A	N/A	N/A
India (2009/10, 2016/17)	31.1 (29.9–32.3)	22.3 (21.4–23.1)	−8.8^¶^	−28.4^¶^	242.8 (229.6–256.0)	207.4 (198.6–216.1)	−35.4^¶^
Mexico (2009, 2015)	56.5 (54.5–58.4)	53.1 (51.7–54.4)	−3.4^¶^	−6.1^¶^	38.7 (34.0–43.4)	46.4 (44.8–48.0)	7.6^¶^
Philippines (2009, 2015)	74.3 (72.4–76.1)	58.6 (55.9–61.2)	−15.7^¶^	−21.1^¶^	45.5 (43.1–47.8)	40.8 (38.1–43.5)	−4.6^¶^
Russia^††^ (2009, 2016)	68.0 (65.8–70.2)	23.1 (20.6–25.7)	−45.0^¶^	−66.1^¶^	76.1 (71.1–81.2)	25.4 (22.6–28.1)	−50.7^¶^
Thailand (2009, 2011)	17.8 (16.5–19.2)	25.7 (23.7–27.8)	7.9^¶^	44.2^¶^	9.1 (8.4–9.9)	13.6 (12.5–14.8)	4.5^¶^
Turkey^§§^ (2008, 2016)	13.3 (12.0–14.6)	17.5 (15.5–19.7)	4.2^¶^	31.8^¶^	6.7 (6.0–7.4)	10.5 (9.2–11.8)	3.7^¶^
Ukraine^††^ (2010, 2016)	46.3 (44.2–48.4)	25.0 (23.2–26.8)	−21.3^¶^	−46.0^¶^	15.8 (14.9–16.7)	8.9 (8.2–9.7)	−6.8^¶^
Uruguay (2009, 2017)	44.3 (42.0–46.5)	34.5 (31.6–37.5)	−9.8^¶^	−22.1^¶^	1.0 (1.0–1.1)	0.9 (0.8–1.0)	−0.1^¶^
Vietnam (2010, 2015)	16.9 (15.8–18.1)	16.0 (14.8–17.3)	−0.9	−5.5	10.8 (10.0–11.6)	11.0 (10.1–11.9)	0.1

**Abbreviations:** CI = confidence interval; N/A = not applicable.

* Population is presented in millions and has been rounded down to the nearest 100,000.

^†^ Both prevalence and population estimates are shown. The estimated differences in population are different from estimated differences in prevalence because of changing population sizes in the 11 countries.

^§^ Percentage change is calculated as [(t2-t1)/t1] x 100 where t1 is the prevalence reported during the first round of GATS and t2 is the prevalence reported during the most recent round.

^¶^ Statistically significant change, p<0.05.

** In 2008, Brazil completed one round of GATS and, in 2013, integrated GATS into its national health survey conducted among adults aged ≥18 years. Thus, Brazil’s data across time compares results for adults aged ≥18 years. Brazil’s 2013 national health survey did not assess the indicators on exposure to secondhand smoke and exposure to advertisements, promotions, or sponsorships in any location.

^††^ In the most recent round, Russia and Ukraine did not survey certain geographic areas that were surveyed in the baseline round.

^§§^ Turkey has completed three rounds of GATS (2008, 2012, and 2016). Data shown are from 2008 as the baseline and 2016 as the latest round.

**FIGURE 1:**
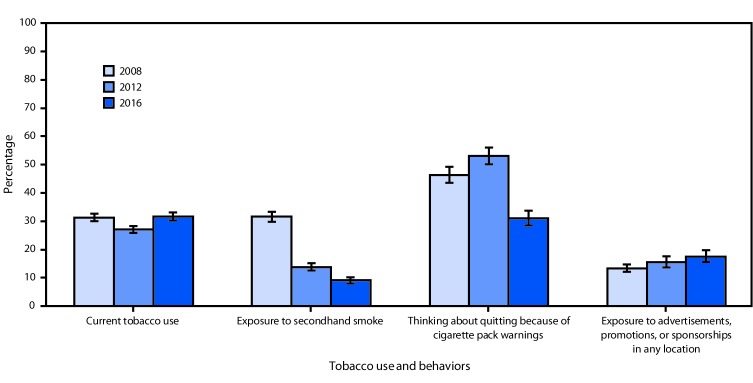
Estimated prevalence of current tobacco use, secondhand smoke exposure, thinking about quitting because of warning labels, and exposure to tobacco advertisements, promotions, or sponsorships among persons aged ≥15 years — Global Adult Tobacco Survey, Turkey, 2008, 2012, and 2016*^,^^†^^,^^§^ * For current tobacco use, secondhand smoke exposure, and thinking about quitting because of warning labels, between surveys in 2008 and 2012, prevalence estimates with p-values <0.05 were considered statistically significant. ^†^ For current tobacco use, secondhand smoke exposure, and thinking about quitting because of warning labels, between surveys in 2012 and 2016, prevalence estimates with p-values <0.05 were considered statistically significant. ^§^ For secondhand smoke exposure, thinking about quitting because of warning labels, and exposure to tobacco advertisements, promotions, or sponsorships, between surveys in 2008 and 2016, prevalence estimates with p-values <0.05 were considered statistically significant.

**FIGURE 2:**
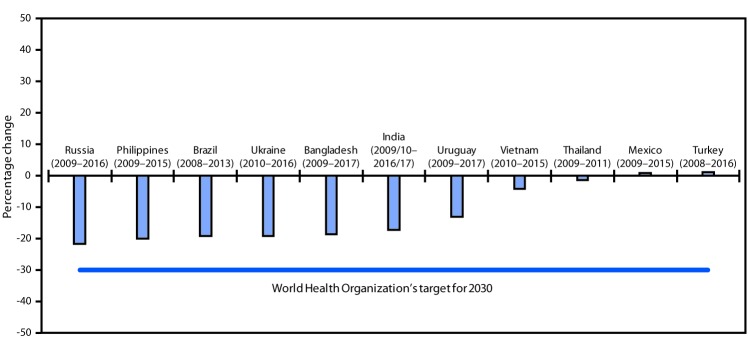
Estimated change in current tobacco use*^,^^†^ prevalence among persons aged ≥15 years — Global Adult Tobacco Survey (GATS), 11 countries,^§^^,^^¶^^,^** 2008–2017 * Current tobacco use is defined as either smoking tobacco or using smokeless tobacco either “every day” or “some days.” ^†^ Percentage change is calculated as [(t2-t1)/t1] x 100 where t1 is the prevalence reported during the first round of GATS and t2 is the prevalence reported during the most recent round. ^§^ Statistically significant change (p<0.05) was noted for Bangladesh, Brazil, India, Philippines, Russia, Ukraine, and Uruguay. ^¶^ In the most recent round of GATS, Russia and Ukraine did not cover certain geographic areas that were covered in the baseline round. ** In 2008, Brazil completed one round of GATS and, in 2013, integrated GATS into its national health survey conducted among adults aged ≥18 years. Thus, Brazil’s data across time compares results for adults aged ≥18 years.

## Discussion

The 11 countries included in this assessment of tobacco use and tobacco-related behaviors are home to 70% of the world’s tobacco users; approximately 2.3 million annual tobacco-attributable deaths occur in these countries (*1*). Although seven of the 11 countries made measurable progress toward WHO’s target of a 30% reduction in tobacco use by 2030, country-level progress varied. As of January 2018, 181 parties had ratified WHO’s FCTC, including all 11 countries highlighted in this report. The ratification of FCTC by these 11 countries demonstrates their commitment to implementing, enforcing, and strengthening tobacco-control efforts, as evidenced by changes in current tobacco use and progress toward WHO’s 2030 target. Continued implementation of MPOWER strategies could help reduce overall tobacco related morbidity and mortality in these countries and worldwide (*3*,*5*,*6*).

Estimated decreases in SHS exposure occurred in seven of the assessed countries. To protect persons from SHS, Article 8 of FCTC encourages signatories to adopt and implement measures that protect persons from SHS exposures in multiple settings (*4*). For all countries with an estimated decline in SHS exposure, the declines were ≥20%, which might reflect the comprehensiveness and enforcement of smoke-free laws. As of 2019, five of the 11 assessed countries had laws that mandated 100% of public places to be smoke-free or had subnational smoke-free legislation that covered at least 90% of the population (*7*).

Significant gains were also made in the proportion of persons considering quitting because of warning labels. Article 11 of FCTC encourages parties to adopt and implement effective measures to ensure that tobacco product packaging and labels do not promote a tobacco product and effectively warn about the dangers of tobacco use (*4*). Currently, all 11 assessed countries have large warnings on their cigarette packages (*7*), with the warnings occupying 30%–85% of the largest package surface. In most countries, the pictorial health warnings were enlarged, text was enhanced, or both (*8*). Adoption of more effective health warnings on tobacco packages (e.g., plain packaging or larger pictorial warnings) could help increase quit attempts (*9*,*10*).

Six countries experienced an estimated decrease in exposure to tobacco advertising, suggesting that gains in protecting persons from exposure to tobacco advertising have been made. Article 13 of FCTC calls for countries to undertake comprehensive bans on tobacco advertising (*4*). As of 2019, four of the 11 assessed countries had bans on all forms of direct and indirect tobacco advertising, resulting in ≥90% of the population being covered by subnational legislation prohibiting tobacco advertising (*7*).

The findings in this report are subject to at least three limitations. First, data were self-reported, which might be subject to misreporting, recall bias, or social desirability bias. Second, the interval between survey rounds varied from 2 to 8 years, which might affect the magnitude of the change in the indicators assessed, given that some countries had more time to implement programs and policies than did others. Finally, the survey did not assess actual policy implementation or level of enforcement.

Progress in reducing tobacco use and addressing tobacco-related behaviors varies across countries. Opportunities exist for countries to improve tobacco control through the implementation and enforcement of evidence-based strategies, which estimates suggest could save 100 million lives by the end of the century (*5*). Continued surveillance of tobacco use, including new and emerging products, and other tobacco-related measures are also critical for informing tobacco control policy, planning, and practice worldwide.

**Summary**

**What is already known about this topic?**

Each year, tobacco use is responsible for approximately 8 million deaths worldwide.

**What is added by this report?**

Analyses of data from 11 countries that conducted at least two rounds of the Global Adult Tobacco Survey showed progress in tobacco control efforts in terms of tobacco use; exposure to secondhand smoke; contemplated quitting because of cigarette package warning labels; and exposure to tobacco advertising, promotions, and sponsorships.

**What are the implications for public health practice?**

Continued implementation and enforcement of proven tobacco control interventions and strategies at the country level, as outlined in the World Health Organization’s MPOWER strategies, can help reduce tobacco use, which is expected to reduce tobacco-related morbidity and mortality.

## Footnotes

* The six components of MPOWER are “monitor” tobacco use and prevention policies; “protect” persons from tobacco smoke; “offer” help to quit tobacco use; “warn” about the dangers of tobacco; “enforce” bans on tobacco advertising, promotion, and sponsorship; and “raise” taxes on tobacco. https://www.who.int/tobacco/mpower/mpower_report_full_2008.pdf.

† https://journals.sagepub.com/doi/full/10.1177/1757975913499800.

§ Second round of GATS data for Brazil were obtained from a national health survey (Pesquisa Nacional Por Amostrade Domicilios), which integrated the entire GATS into the survey, thus producing nationally representative estimates for adults aged ≥18 years. All analyses for Brazil are among adults aged ≥18 years. Turkey has three rounds of GATS data (2008, 2012, and 2016).

¶ In most countries in this analysis, tobacco that is smoked is the predominant type of tobacco used. This is not the case in India and Bangladesh, where smokeless tobacco is the predominant type of tobacco used.

** Operational definition for persons who currently use tobacco can be found in GATS Indicator Definitions, Version 2.1. Current tobacco use is defined as having responded “daily” or “less than daily” to either “Do you currently smoke tobacco on a daily basis, less than daily, or not at all?” and “Do you currently use smokeless tobacco on a daily basis, less than daily, or not at all?” https://nccd.cdc.gov/GTSSDataSurveyResources/Ancillary/DownloadAttachment.aspx?ID=53.

†† Operational definition for secondhand smoke exposure can be found in GATS Indicator Definitions, Version 2.1. Secondhand smoke exposure in a public place is defined as having responded “yes” to any of the four following questions: “Did anyone smoke inside of any government building or government offices that you visited in the past 30 days?,” “Did anyone smoke inside of any health care facilities that you visited in the past 30 days?,” “Did anyone smoke inside of any restaurants that you visited in the past 30 days?,” or “Did anyone smoke inside of any public transportation that you used in the past 30 days?” https://nccd.cdc.gov/GTSSDataSurveyResources/Ancillary/DownloadAttachment.aspx?ID=53.

§§ Operational definition for thinking about quitting because of warning labels can be found in GATS Indicator Definitions, Version 2.1. Contemplated quitting because of warning labels is defined as current tobacco smokers, those respondents responding “daily” or “less than daily” to “Do you currently smoke tobacco on a daily basis, less than daily, or not at all?,” who responded “yes” to “In the last 30 days, did you notice any health warnings on cigarette packages?” and “In the last 30 days, have warning labels on cigarette packages led you to think about quitting?” https://nccd.cdc.gov/GTSSDataSurveyResources/Ancillary/DownloadAttachment.aspx?ID=53.

¶¶ Operational definition for awareness of cigarette advertising promotion, and sponsorships can be found in GATS Indicator Definitions, Version 2.1. Being aware of cigarette advertising and promotions is defined as responding “yes” to any of the four following questions: “In the last 30 days, have you noticed any advertisements or signs promoting cigarettes in the following places? A) In stores where cigarettes are sold?; B) On television?; C) On the radio? D) On billboards? E) On posters? F) In newspapers or magazines? G) In cinemas? H) On the internet? I) On public transportation vehicles or stations? J) On public walls? K) Anywhere else?,” “In the last 30 days, have you noticed any sport or sporting event that is associated with cigarette brands or cigarette companies?,” or “In the last 30 days, have you noticed any of the following types of cigarette promotions? A) Free samples of cigarettes? B) Cigarette at sale prices? C) Coupons for cigarettes? D) Free gifts or special discount offers on other products when buying cigarettes? E) Clothing or other items with a cigarette brand name or logo? F) Cigarette promotions in the mail?” The exact questions asked in the survey varied depending on country circumstances. In India, the definition covers both cigarettes and bidis instead of cigarettes only. https://nccd.cdc.gov/GTSSDataSurveyResources/Ancillary/DownloadAttachment.aspx?ID=53.

## References

[1] GBD 2017 Risk Factor Collaborators. Global, regional, and national comparative risk assessment of 84 behavioural, environmental and occupational, and metabolic risks or clusters of risks for 195 countries and territories, 1990–2017: a systematic analysis for the Global Burden of Disease Study 2017. Seattle, WA: Institute for Health Metrics and Evaluation; 2018. 10.1016/S0140-6736(18)32225-6 30496105 PMC6227755

[2] World Health Organization. WHO report on the global tobacco epidemic, 2017: monitoring tobacco use and prevention policies. Geneva, Switzerland: World Health Organization; 2017. https://apps.who.int/iris/bitstream/handle/10665/255874/9789241512824-eng.pdf;jsessionid=5B12F0106C9C5146FD02389C555F41F2?sequence=1

[3] World Health Organization. WHO report on the global tobacco epidemic, 2008: the MPOWER package. Geneva, Switzerland: World Health Organization, 2008. https://www.who.int/tobacco/mpower/mpower_report_full_2008.pdf

[4] World Health Organization. WHO framework convention on tobacco control. Geneva, Switzerland: World Health Organization; 2005. https://www.who.int/tobacco/framework/WHO_FCTC_english.pdf

[5] Frieden TR, Bloomberg MR. How to prevent 100 million deaths from tobacco. Lancet 2007;369:1758–61. 10.1016/S0140-6736(07)60782-X 17512860

[6] Henning K. Working together around the world to kick the big tobacco habit. Bethesda, MD: Project HOPE; 2017. https://www.healthaffairs.org/do/10.1377/hblog20170105.058236/full/

[7] World Health Organization. WHO report on the global tobacco epidemic, 2019: offer help to quit tobacco use. Geneva, Switzerland: World Health Organization; 2019. https://apps.who.int/iris/bitstream/handle/10665/326043/9789241516204-eng.pdf?ua=1

[8] Campaign for Tobacco-Free Kids. Tobacco control laws. Washington, DC: Campaign for Tobacco-Free Kids; 2019. https://www.tobaccocontrollaws.org

[9] Azagba S, Sharaf MF. The effect of graphic cigarette warning labels on smoking behavior: evidence from the Canadian experience. Nicotine Tob Res 2013;15:708–17. 10.1093/ntr/nts194 22990228

[10] Borland R, Yong HH, Wilson N, How reactions to cigarette packet health warnings influence quitting: findings from the ITC four-country survey. Addiction 2009;104:669–75. 10.1111/j.1360-0443.2009.02508.x 19215595 PMC4394051

